# Deep subsurface mine stalactites trap endemic fissure fluid Archaea, Bacteria, and Nematoda possibly originating from ancient seas

**DOI:** 10.3389/fmicb.2015.00833

**Published:** 2015-08-11

**Authors:** Gaëtan Borgonie, Borja Linage-Alvarez, Abidemi Ojo, Steven Shivambu, Olukayode Kuloyo, Errol D. Cason, Sihle Maphanga, Jan-G Vermeulen, Derek Litthauer, Colin D. Ralston, Tullis C. Onstott, Barbara Sherwood-Lollar, Esta Van Heerden

**Affiliations:** ^1^Extreme Life IsyensyaGentbrugge, Belgium; ^2^Department of Biotechnology, University of the Free StateBloemfontein, South Africa; ^3^Geology, Beatrix Gold MineTheunissen, South Africa; ^4^Bergville Retirement VillageGeorge, South Africa; ^5^Department of Geosciences, Princeton UniversityPrinceton, NJ, USA; ^6^Department of Earth Sciences, University of TorontoToronto, ON, Canada

**Keywords:** subsurface sea, stalactites, diversity, *Monhystrella parvella*

## Abstract

Stalactites (CaCO_3_ and salt) from water seeps are frequently encountered in ceilings of mine tunnels whenever they intersect water-bearing faults or fractures. To determine whether stalactites could be mineralized traps for indigenous fracture water microorganisms, we analyzed stalactites collected from three different mines ranging in depth from 1.3 to 3.1 km. During sampling in Beatrix gold mine (1.4 km beneath the surface), central South Africa, CaCO_3_ stalactites growing on the mine tunnel ceiling were collected and observed, in two cases, to contain a living obligate brackish water/marine nematode species, *Monhystrella parvella*. After sterilization of the outer surface, mineral layers were physically removed from the outside to the interior, and DNA extracted. Based upon 16S and 18S rRNA gene sequencing, Archaea, Bacteria, and Eukarya in different combinations were detected for each layer. Using CT scan and electron microscopy the inner structure of CaCO_3_ and salt stalactites were analyzed. CaCO_3_ stalactites show a complex pattern of lamellae carrying bacterially precipitated mineral structures. Nematoda were clearly identified between these layers confirming that bacteria and nematodes live inside the stalactites and not only in the central straw. Salt stalactites exhibit a more uniform internal structure. Surprisingly, several Bacteria showing highest sequence identities to marine species were identified. This, together with the observation that the nematode *M. parvella* recovered from Beatrix gold mine stalactite can only survive in a salty environment makes the origin of the deep subsurface colonization enigmatic. The possibility of a Permian origin of fracture fluids is discussed. Our results indicate stalactites are suitable for biodiversity recovery and act as natural traps for microorganisms in the fissure water long after the water that formed the stalactite stopped flowing.

## Introduction

Studying deep subsurface terrestrial biodiversity can only be achieved through deep drilling and mining or exploration of caverns. In deep mining, access to fracture water is unpredictable, irregular and the volume available for collection may be too limited. The latter is important as cell counts of bacteria in deep subsurface fracture water is typically low, requiring large volumes for deriving sufficient DNA for characterization of the taxonomic diversity. During deep mine sampling, we have encountered on several occasions stalactites on the corridor or tunnel ceilings.

In many caverns a large variety of communities comprised of Algae, Bacteria, and Fungi are found embedded in stalactites and in some cases, these microorganisms have been implicated in the precipitation of carbonate soda-straw (“straws”) stalactites in caves. This occurs through a number of processes that include ammonification, denitrification, sulfate reduction and anaerobic sulfide oxidation (Danielli and Edington, 1983; Cox et al., 1989; Castanier et al., 1999, 2000; Riding, 2000; Desmarchelier et al., 2006). Thus, underground mine stalactites raised the possibility that they may act as natural traps for microorganisms in the fissure water. More importantly they could serve as enrichment sites allowing sampling drip waters that defy more traditional sampling methods due to the low water volume. Arguing against such an approach is the contamination issue of a stalactite hanging free in a strongly ventilated mine corridor where anthropogenic contamination is a daily possibility. Previous research on fungal spores in mine air (Pohl et al., 2007) justified this concern and biofilms growing on tunnels walls below a fissure water outlet from a fracture or a borehole is generally considered not to be representative of the microbial community with the fracture zone that is supplying the water. Furthermore, the small size of the stalactites and the enrichment potential would only allow studying partial biological/chemical content.

During routine sampling at a depth of −1.4 km in Beatrix gold mine, South Africa, several stalactites were collected and stored in Ziplock sample bags. Upon examination with a stereomicroscope we identified a nematode in the fluid that came out of these stalactites. In the course of the following days, a total of eight nematodes were observed in the sampling bag. This puzzling observation led us to examine several stalactites from three different mines to test the hypothesis that stalactites in mines may serve as useful natural (enrichment) traps for Archaea, Bacteria, and Eukarya. Additionally we wanted to establish the origin of the nematodes that occurred within the stalactites, as they are not known to be tube builders compared to other Animalia.

## Materials and methods

### Origin of the stalactites

The Republic of South Africa hosts some of the world's deepest mines, some of which exceed 4 km below land surface (kmbls). These, deep gold, platinum and diamond mines and their network of tunnels and crosscuts, allow exceptional access to the deep subsurface. During the course of normal mining operations, the advancing tunnels or exploratory boreholes intersect water-bearing fractures. Stalactites occur associated with water-weeping fractures intersected by the tunnel. Since the presence of stalactites in the mines is haphazard, stalactites were collected from mines when and where available. Samples were taken at Beatrix gold mine, Moab Khotsong gold mine, Evander gold mine and Tau Tona gold mine.

#### Beatrix gold mine (Sibanye Gold, Ltd.) 28°14′24.37″S/26°47′49.30″E

Beatrix gold mine is located near the towns of Welkom and Virginia, some 240 km southwest of Johannesburg in the Free State Province of South Africa. Geologically the mine is located along the southern margin of the Witwatersrand Basin. Twelve carbonate stalactites were collected in the same tunnel at two different locations approximately 100–200 m apart on level 26 of #3 shaft, 1.4 km below the surface. The stalactites were collected in 2009–2010 and 2013. During the 2010 collection campaign, five soil samples were taken from the tunnel floor to look for nematodes. This tunnel is located in the Witwatersrand Supergroup, which at this location is directly overlain by 400–800 m of Carboniferous Karoo sediments. The age of the carbonate stalactites is unknown but cannot be older than 2–3 years since the tunnel was excavated in 2007.

#### Moab Khotsong, (Anglogold Ashanti Ltd.) 26°59′14.20″S/26°48′5.01″E

Moab Khotsong is the newest deep-level gold mine in South Africa and is situated near Orkney, Klerksdorp, and Viljoenskroon in North West Province, about 180 km southwest of Johannesburg. The principal reef is the Vaal Reef of which the gold grade and morphology are considered to be a down-dip extension to the south and southeast of Kopanang and Great Noligwa mines. The reef is comprised of an oligomictic conglomerate, where gold is associated with carbon at the base. Stalactite samples some as large as 3 m were collected in December 2012 growing on the rim of a man-made ventilation shaft at level 120, which is at a depth of −3.1 km. The age of these stalactites can be no more than 5 years for the smallest used for DNA analysis, and up to 10 years for the largest specimen used in the CT scan.

#### Evander gold mine (Harmony Gold Mining Company Ltd.) 26°27′20.57″S/29°4′15.76″E

Evander gold mine is situated 8 km northwest from Secunda in the Mpumalanga Province. The Evander Basin is a tectonically preserved sub-basin outside the main Witwatersrand Basin and forms an asymmetric syncline, plunging north-east. It is structurally complex with a series of east-north-east striking normal faults. At the southeast margin of the basin, vertically to locally overturned reef is present. The only economic reef horizon exploited in the Evander Basin is the Kimberley Reef. The Intermediate Reef is generally poorly mineralized, except where it erodes the sub-cropping Kimberley Reef to the south and west of the basin. Several small dried out stalactites were collected in 2007 at shaft #8, level 13 at a depth of ~1.4 km. One of these was kept in a closed tube from 2007 until 2012 when it was used for the present analysis. The age of the stalactite is unknown.

#### Tau Tona gold mine (Anglo Gold Ashanti Ltd.) 26°21′21.29″S/27°24′10'12″E

Tau Tona gold mine is located south of Carletonville in the Gauteng Province and about 70 km southwest of Johannesburg. The sampling site was a nearly horizontal borehole located on level 118 at a depth of 3.4 km in the Witwatersrand Supergroup and intersected the Pretorious fault zone. The borehole was not sealed and flowed intermittently. This borehole was the site of the deepest metazoan ever found in 2011, a monhysterid nematode of which only a DNA sequence was known to date. The fissure water was sampled again in 2012–2013 in an attempt to recapture the nematode previously found there because of its relevance to the DNA results from the Beatrix gold mine subsurface nematode.

### Stalactites used for analysis

Twelve CaCO_3_ stalactites were collected in Beatrix gold mine of which two contained a total of 20 nematodes. Of those 12 stalactites, two containing nematodes and one devoid of nematodes was used for micro-CT scan, XRD and DNA analysis. Only one still intact stalactite from Evander mine was used for XRD and DNA analysis. One small stalactite from Moab Khotsong was used for XRD analysis. One very large salt stalactite from Moab Khotsong was used for macro CT scan and DNA analysis. The different stalactite layers are designated as NSX.Y where N is the first letter of the mine from which the stalactite (S) was collected, X is the number identifying the collection order of the stalactite in a given mine, Y refers to the peeled or dissolved layer in question counting from the outside toward the center. For example, ES1.4 means the first collected stalactite from Evander mine, layer number 4.

### Micro-CT scan

Micro-CT is a non-destructive 3D imaging technique for studying the internal structure of an object. Setup and conditions are described in detail in Borgonie et al. (2010). Volume rendering and segmentation was performed using VGStudio Max (Volume Graphics). In an attempt to visualize the nematodes, the bulk was rendered transparent. Three stalactites were scanned. One yielded nematodes while the other two did not.

### Macro-CT scan

Larger stalactites were CT scanned at the UFS Campus Netcare Hospital using a General Electric HD 750 (Milwaukee, USA) with following settings: KvP 140, MA 450 helical with 0.625 mm slices with a 0.312 interval.

### Scanning electron microscopy

Pieces of stalactite were mounted on a specimen stub and sputter coated with gold using a BIO-RAD (Microscience Division) Coating System (London, UK) and observed with a Jeol JSM 8440 SEM microscope. For fixation of the CaCO_3_ containing nematodes, 70°C 30% formaldehyde was used followed longitudinal cutting with a sterile scalpel, then dehydration, critical point drying, coating and mounting. SEM pictures were assembled in plates using Adobe Photoshop (Adobe Systems Inc., San Jose, CA, USA) and where relevant colored using Smart Photo editor (Anthropics Technology, Ltd., London, UK).

### X-ray diffraction (XRD)

Before melting the different stalactites a small piece was removed and homogenized from each for XRD analysis. The samples were ground to a grain size of < 50 μm using a carbon steel ring and agate mortar and pestle. The loose powder samples were then mounted into steel ring sample holders, and slightly compressed to give a smooth surface. XRD measurements were performed using a Panalytical Empyrean X-ray diffractometer, with the following con urations: The samples are irradiated with CuKα-radiation generated at 40 mA and 45 kV. No monochromator was inserted while a fixed divergence slit of 0.2177° was part of incoming beam optics. Scanning was continuous starting at 5° 2θ and ending at 70° 2θ, with step size 0.017 2θ for 15.875 s per step. Measurements were carried out on a stationary sample without spinning of the sample holder. The identification of minerals was carried out using Panalytical Highscore software with the PDF-2 (2009) powder diffraction file database, and estimates of relative abundances were made based on semi-quantitative data associated with the files in this database.

### Dissolving salt stalactites/dissecting carbonate stalactite

The outer surface of salt/carbonate stalactites was sterilized by immersion in 100% ethanol and set alight allowing the ethanol to burn off. Three small pieces were subsequently chipped off and inoculated onto three different media namely, Luria Bertani (LB), nutrient agar (NA), and Potato dextrose Agar (PDA) to confirm the effectiveness of sterilization. The individual layers within the stalactite in the case of carbonate were carefully dissected using sterilized tweezers; pieces were collected in 1.5 mL Eppendorf tubes for DNA extraction. For the salt stalactites sterile deionized water was sprayed on the layers to dissolve the stalactite carefully. Cross contamination through dripping from the ends of the sample was prevented by holding the stalactite horizontally along its longest axis and spraying carefully from beneath.

### Nematode culture/halotolerance test

Collection and identification of nematodes from fluids that leached out of the Beatrix gold mine carbonate stalactite was done aseptically. Twelve nematodes were recovered in the course of 7 days in 2010, and the second stalactite collected in 2013 yielded eight nematodes. Culturing was attempted by placing the nematodes on a 3% salt-agar plate covered with the saline (14 g/L) water that oozed out of the stalactite. Halotolerance by the nematodes was performed in artificial salt agar. Due to the low number of nematodes available, the test was performed twice using 12 nematodes in total. The initial salt concentration was 14 g/L; nematodes were transferred to a different concentration every 24 h. The nematodes died quickly when the salt concentration was too high/low acting as a suitable marker for tolerance. Control nematodes: *Halicephalobus mephisto* collected previously from the same mine but from a borehole (Borgonie et al., 2011) and *Poikilolaimus oxycercus* a surface collected nematode. For both *P. oxycercus* and *H. mephisto* thirty worms were used and were directly placed on agar plates with different salt concentrations. In an attempt to identify Bacteria used as food by *M. hystrella* one specimen was slowly dissolved in a 1% NaOCl solution until the outer cuticle had dissolved as observed under a stereomicroscope. The specimen was then washed three times in deionized water and DNA extracted.

## DNA analysis

### DNA extraction

#### Nucleic acid extraction from the stalactite layers

CaCO_3_stalactite layers were pulverized in an Eppendorf tube using a sterile pestle. The dissolved halite stalactite layers were collected in 50 mL Falcon tubes and then filtered using a 0.22 μm filter membrane (MIS). DNA was extracted from the pulverized CaCO_3_ and filter using the InstaGene Matrix kit (BIO-RAD 732-6030). Quality and concentration of DNA was determined using ND-1000 Spectrophotometer (NanoDrop).

#### Nucleic acid extraction from nematodes

Nematodes were collected from the petri dish with a sterile platinum needle and transferred into sterile deionized water in a 1.5 mL Eppendorf tube. DNA was extracted directly using the InstaGene Matrix kit (BIO-RAD 732-6030). Quality and concentration of DNA was determined using ND-1000 Spectrophotometer (NanoDrop).

### PCR amplification

#### PCR amplification from stalactites

Fragments of 16S and 18S rDNA suitable for DGGE analysis were obtained by using different primer sets for different domains, (Muyzer et al., 1993; Casamayor et al., 2002; Mühling et al., 2008; Stomeo et al., 2012), Bacteria: 27F, 341FGC, 908R, and 1492R; Archaea: 934R and 344FGC; Eukarya: 516R and 1AFGC. The primer sets and the PCR conditions were modified from protocols described by Casamayor et al. (2002). Due to the small size of the stalactites, an initial PCR was performed using 27F and 1492R primers followed by *nested* PCR using 341F with GC clamp and 908R. PCR conditions for the two steps were: initial denaturation at 94°C for 3 min; 29 cycles (94°C, for 30 s; 55°C for 30 s; 72°C for 1 min); and a final extension at 72°C for 5 min. For the Archaeal 16S rDNA fragments, we used an initial denaturation step at 94°C for 2 min followed by 30 PCR cycles with annealing at 61°C and a final 10 min. extension step at 72°C. The eukaryal 18S rDNA fragments were amplified using: initial denaturation at 94°C for 130 s; 35 cycles (94°C for 30 s; 57°C for 30 s; 72°C for 7 min); and a final extension at 72°C for 5 min.

#### PCR amplification of 18S rRNA gene from nematodes in culture

PCR amplification of 18S rDNA fragments was performed using primer setsEukA [AACCTGGTTGATCCTGCCAGT] and EukB [TGATCCTTCTGCAGGTTCACCTAC] (Diez et al., 2001), and Nem18S(F) [CGCGAATRGCTCATTACAACAGC] and Nem18S(R) [GGGCGGTATCTGATCGCC] (Floyd et al., 2005). A standard reaction volume was 25 μL containing: 1x concentration of Standard *Taq* buffer (New England BioLab), (including 1.5 mM MgCl_2_), 0.5 μM of each primer, dNTPs at a concentration of 0.2 mM for each nucleotide, 0.025 units/μL *Taq* DNA polymerase and 10 ng of extracted DNA template was added to each reaction. The thermocycling conditions used for eukaryal and nematode PCR reactions are: an initial denaturation at 94°C for 5 min; 35 cycles (94°C for 30 s; 54°C for 30 s; 72°C for 1 min); and a final extension at 72°C for 10 min (Floyd et al., 2005) in a PXE 0.2 Thermal Cycler (Thermo Electron Corporation).

#### PCR amplification of the nematode mitochondrial COI gene

A portion of the mitochondrial cytochrome oxidase c subunit 1 (COI) gene was amplified with the primer sets COI1F (5′-GGT CAA CAA ATC ATA AAG ATA TTG G-3′) and COI1R (5′-TAA ACT TCA GGC TGA CCA AAA AAT CA-3′) (Valenzuela et al., 2007); and JB3F (5′-TTT TTT GGG CAT CCT GAG GTT TAT-3′) and JB4.5R (5′-TAA AGA AAG AAC ATA ATG AAA ATG-3′) (Derycke et al., 2005). Amplification was conducted for 35 cycles, each consisting of a 30 s denaturation at 94°C, 30 s annealing at 54°C, and 30 s extension at 72°C, with an initial denaturation step of 5 min at 94°C and a final extension step of 10 min at 72°C.

### DGGE analysis

#### DGGE analysis of PCR products of ribosomal DNA fragments from stalactites

The amplified 16S/18S rRNA gene fragments (600–800 ng) were separated on 7% (w/v) polyacrylamide gel with a urea/formamide denaturing gradient ranging from 40 to 60%. Electrophoresis was performed in 1X TAE buffer (40 mM Tris, 20 mM acetic acid, 1 mM EDTA; pH 8.3) at a constant voltage of 100 V at 60°C and run for 16 h. Gels were stained for 40 min in 1X TAE buffer containing Sybr SYBR® Gold Nucleic acid stain (Molecular Probes, Invitrogen) and visualized with UV radiation by using a Gel doc XR and the Quantity one 4.6.7 imaging software (Bio-Rad).

### Sequencing of DNA fragments

#### Sequencing of stalactites DNA fragments

DNA was recovered from each excised band reamplified using the same set of primers mentioned above, without the GC clamp. The PCR products were then purified using Exonuclease I (Exo I) Thermo Scientific FastAP and Thermosensitive Alkaline Phosphatase (#EF0651, Werle et al., 1994). Sequencing reactions were performed with the ABI Prism™Big Dye terminator™V3.1 cycle sequencing ready reaction kit and data collected on an ABI 3130*XL* genetic analyzer (Applied biosystems). The sequences were checked for chimeras using Bellerophon (Huber et al., 2004) and then compared to the GenBank nucleotide database [National Center for Biotechnology Information, (NCBI)] using BLAST. Neighbor-joining phylogenetic trees were generated using reference sequences from the RDP (Maidak et al., 1997) and using Geneious 4.8.5.

#### Sequencing of nematode DNA fragments

The amplified DNA fragments were sequenced using the ABI Prism™BigDye terminator™V3.1 cycle sequencing kit and data collected on an ABI 3130*XL* genetic analyzer (Applied biosystems). The quality of ABI files retrieved using FinchTV software was evaluated and the sequence reads were assembled using CodonCode Aligner software (CodonCode Corporation, Dedham, MA, USA). Overlapping reads or contigs thar represented the consensus regions of DNA were aligned using ClustalW (http://www.ebi.ac.uk/Tools/msa/clustalw2/). The alignment was examined for conserved regions so as to provide sufficient information that reveals any similarity between the two isolated organisms. BLASTN analysis of DNA database was used. Sequences were compared to the Nucleotide collection (nr/nt) database and optimized for highly similar sequences (Megablast).

#### Genbank submissions

All sequences were deposited in Genbank with accession numbers: Bacteria KJ546057-KJ546071, Archaea KJ546072-KJ546075, Eukarya: KJ546076-KJ546081.

#### Microscopy

The low number of nematodes available made a tradeoff necessary between retaining enough specimens to measure for taxonomic purposes and retaining enough for attempted culturing. To avoid losing too many nematodes to the attempted culture only three adult nematodes were used for taking microscopic measurements. These nematodes were anesthetized by a brief slide heating. A Zeiss Axioskop Microscope was used. This approach had the disadvantage that not all small details could be measured but left enough valid measurements for proper identification of the nematode species. These selected nematodes were returned to the culture but were not used for the salt tolerance experiment.

## Results

XRD analysis of the carbonate stalactites from Beatrix and Evander showed its composition to be mixture of calcite and aragonite. The XRD analysis of the Moab Khotsong salt stalactite determined the composition as ~98% halite +~1% sylvite +~1% quartz.

The CaCO_3_ stalactites each yielded 3–4 layers when peeled, whereas the salt stalactite from Moab Khotsong was dissolved in four layers (Table 1). After sterilization none of the outer layers that were tested on PDA, LB or NA agar showed any growth indicating that sterilization worked satisfactorily.

Table 1**Archaeal, Bacterial, and Eukaryal DNA results from the stalactite extractions**.

**Table:** 

**Layer**	**# DGGE bands**	**DNA (ng/μl)/260/280 ratio**	**Closest relative/Taxonomic position**	**Acc. number**	**% Identity**	**Source**
**ARCHAEA/BACTERIA**						
E S1.1	2	7.43/1.74	*Ureibacillus*	JQ734423	100	Oil-water mixture
E S1.2	1	8.23/1.61	*Klebsiella*	KC907123.1	100	Soil
	1		*Uncultured Citrobacter*	GQ416126	100	Biological degreasing systems
E S1.3	2	5.82/1.54	*Pantoea*	AJ002811	100	Gut
E S1.4	2	5.62/1.80	*Thermus*	AB661716	100	Deep mine, Beatrix
B S2.1	2	147.13/1.70	*Bowmanella*	GQ246642	99	Ocean water
B S2.2	2	41.02/1.74	*Klebsiella*	HE716897	100	Host leaves
B S2.3	1	13.45/2.22	*Uncultured SGUS738 (Oceanospirillales)*	FJ202645	91	Caribbean coral
	1		*Staphylococcus* sp.	EF419336	100	Soil
B S5.1	0	19.99/1.80				
B S5.2	1	10.04/1.98	Could not be sequenced			
B S5.3	0	6.54/1.79				
B S6.1	1	13.36/1.57				
B S6.2	1	28.25/1.54	*Cupriavidus*	DQ777727	100	Soil
B S6.3	1	16.79/1.85	*Cupriavidus*	DQ777727	100	Soil
M S3.1	1	10.0/2.04	*Archaeon clone (Halobacteriales)*	DQ336966.1	99	Deep mine Evander
	3		*Alcanivorax* sp.	HQ662960	99	Sea water
M S3.2	1	7.74/1.70	*Archaeon clone (Halobacteriales)*	DQ336966.1	99	Deep mine Evander
	1		*Alcanivorax* sp.	HQ662960	100	Sea water
	1		*Uncultured Bacteroidetes*	JF421218	98	Saline-alkali soil
M S3.3	1	3.63/1.97	*Archaeon clone (Halobacteriales)*	DQ336966.1	99	Deep mine Evander
	1		*Pantoea* sp.	AF394539	100	Grass
M S3.4	1	3.72/1.94	*Archaeon clone (Halobacteriales)*	DQ336966.1	99	Deep mine Evander

**Table:** 

**Layer**	**# DGGE bands**	**Closest relative/Taxonomic position**	**Acc. number**	**% Identity**	**Source**
**EUKARYA**					
E S1.1	2	Could not be sequenced			
E S1.2	3	*Acanthamoeba (Protozoa)*	GQ397466.1	99	Fresh water
	1	*Candida (Fungi)*	HM161746.1	92	Human associated
E S1.3	2	*Uncultured eucarya*	FN394866.1	92	
E S1.4	4	Could not be sequenced			
B S2.1	2	Could not be sequenced			
B S2.2	0				
B S2.3	2	Could not be sequenced			
B S5.1	2	Could not be sequenced			
B S5.2	4	*Colpoda (Protozoa)*	JN251157.1	100	Freshwater
		*Uncultured eucarya*	AY905498.1	100	
B S5.3	4	Could not be sequenced			
B S6.1	0				
B S6.2	0				
B S6.3	1	Could not be sequenced			
M S3.1	9	Could not be sequenced			
M S3.2	9	Could not be sequenced			
M S3.3	4	Could not be sequenced			
M S3.4	7	Could not be sequenced			
B S2&5^*^		*Monhystrella parvella (Nematoda)*			Brackish water/thermal springs

*Name of the mine is referred as E (Evander), B (Beatrix), and M (Moab Khotsong). Beatrix stalactite BS 1 and BS2 contained nematodes, BS 3 did not. The listed 260/280 ratio is used to assees purity of the extracted DNA using a Nanodrop® spectrophotometer. A value of ~1.80 is generally accepted as “pure” for DNA*.

* *Isolated by capturing the fluid flowing out of the collected stalactite*.

DNA extraction was successful for all layers and resulted in archaeal, bacterial and/or eukaryal DNA (Table 1). A difference is noted between salt and CaCO_3_ stalactites where the salt stalactite show a more uniform yield of archaeal, bacterial or eukaryote DNA in all layers of the same stalactite, whereas the CaCO_3_ stalactites showed a more variable pattern with not all layers of the same stalactite having the same DNA content. Sequencing of extracted DNA was not always successful (Table 1). Salt stalactites consistently yielded a higher number DNA bands on DGGE than CaCO_3_ stalactites, but yielded poorer sequencing results (Table 1).

Eukaryal sequences revealed one Fungus (Ordo *Saccharomycetales Candida* sp., Berkh, 1923) and a protozoan, *Acanthamoeba* sp. Castellani, 1930 and a ciliate, *Colpoda* sp. Müller, 1773 No nematode DNA was identified in the stalactite layers (Table 1).

Identification of the nematode species that flowed out of the stalactites was done using light microscopy of three adult individuals (Table 2). Notwithstanding the limitations in usable specimen (see Materials and Methods) the nematode species recovered from the two stalactites belong to the genus *Monhystrella* Cobb, 1918 based on the cephalic setae, small body length, and fovea position, presence of a pharyngeal bulbus, a prominent progaster, and vulva at midbody. Measurements were compared with known populations from Ethiopia, Namibia and Bulgaria and confirm the identity as *Monhystrella parvella* (Filipjev, 1931; Jacobs, 1987) a brackish water/marine nematode. The measurements agree with the Namibian population more than the Ethiopian/Bulgarian populations (Table 2). An 18S rRNA gene sequence for *M. parvella* was obtained but could not be compared with the Ethiopian, Namibian or Bulgarian population as no DNA of those samples has been analyzed. Similarity searches using BLASTN algorithm (NCBI database) revealed a 91% similarity to the next closest surface nematode *Diplolamelloides* sp., which is a marine nematode. This indicates that the Beatrix stalactite nematode species has not yet been barcoded on the surface to date. Similarity searches also revealed a 96% identity with a previously collected subsurface nematode DNA at Tau Tona mine in fissure water of 48°C, salinity 14g/L at −3.4 km, 230 km to the North of Beatrix gold mine (Borgonie et al., 2011). An attempt was made to recapture the Tau Tona nematode in 2013, which resulted in two nematode specimens being collected. DNA analysis only was hence possible and revealed a 100% match of the 18S rRNA gene sequence and of the COI mitochondrial gene sequence between Beatrix gold mine *M. parvella* and the newly collected Tau Tona Monhysterid. Sequence comparison between the Tau Tona 2011 and 2013 sequences confirmed the 96% identity of the 18s rRNA gene sequence.

**Table 2:** **Measurements of ***M. parvella*** from Beatrix stalactites compared with the population from Ethiopia, Namibia, and Bulgaria**.

		**Filipjev's specimen from Ethiopia 1926**				**Gerlach's specimen from Bulgaria 1951**			**Beatrix gold mine republic of South Africa 2013**			**Namibia 1986**					
													**Ai-Ais**			**Namutoni**	
	***n***	**Mean**	**Min-max**	***s***	***n***	**Mean**	**Min-max**	***n***	**Mean**	**Min-max**	***s***	***n***	**Mean**	**Min-max**	***n***	**Mean**	**Min-max**
L	11	445.0	396.0–486.0	26.8	7	420.0	380.0–460.0	3	449.6	401.0–493.0	6.2	10	438	427–478	6	472	406–505
v.b.w	11	24.0	21.0–31.0	3.3	7	17.5		3	27.6	22.0–33.0	5.5	10	15.9	14.9–17.0	6	17.1	15–17
ph.l	12	88.0	76.0–101.0	7.0	7	68.9		3	91.3	81.0–103.0	11.0	10	79	72–83	6	91	80–99
t.l.	11	96.0	80.0–117.0	10.5	7	101.2		3	102.6	88.0–120.0	16.1	10	106	94–112	6	112	101–120
v	11	249.0	224.0–271.0	15.8	7	220.5		3	252.6	228.0–276.0	24.0						
Ph.v.l.	11	148.0	133.0–162.0	10.4	7	151.6		3	150.0	134.0–164.0	15.0						
v.a.l	11	100.0	82.0–124.0	10.9	7	98.3		3	109.0	97.0–119.0	11.1	10	106	93–113	6	115	94–123
h.d	12	8.5	7.6–9.3	0.5				3	8.6	8.0–9.4	0.7	10	5.3	5–6	6	5.8	5.5–6.0
f.d.	12	2.9	2.2–3.3	0.3				3	2.6	2.5–2.7	0.1	10		2.4–2.6	6		2.4–2.6
a.f.b.l.	12	9.2	7.5–11.4	1.1				3	9.5	9.2–10.0	0.4						
c.b.w.	12	21.1	19.0–25.0 1.7					3	22.6	20.0–25.0	2.5						
r.l.	11	10.4	7.7–13.2	1.7				3	10.3	8.0–11.0	4.3	10	10.9	10–11.5	6	12.6	11.5–13.5
a	11	18.2	15.0–20.0 1.7		7	24.0	21.0–27.0	3	18.3	16.0–21.0	2.5	10	27.5	23.8–29.5	6	27.6	27.1–28.9
b	11	5.1	4.6–5.4	0.3	7	6.1	5.4–6.8	3	4.8	4.7–4.9	0.1	10	5.5	5.2–6.0	6	5.2	5.1–5.4
c	11	4.7	4.0–5.3	0.4	7	4.2	3.9–4.4	3	4.4	4.1–4.6	0.3	10	4.2	4.0–4.4	6	4.2	4.0–4.4
V	11	55.9	51.0–59.0 2.0		7	52.3	50.0–55.0	3	55.3	55.0–56.0	0.6						
G	7	21.1	17.0–29.0 5.1					1	30.0								
n.r.	11	57.1	51–62.0	3.6				3	57.0	55.0–60.0	2.6						
ph.v.l./ph.l.	11	1.7	1.5–1.9	0.1	7	2.2		3	1.64	1.6–1.7	0.1						
v.a.l./ph.l.	11	1.1	0.9–1.3	0.1	7	1.4		3	1.2	1.1–1.2	0.1						
t.l./ph.l.	11	1.1	0.9–1.3	0.1	7	1.5		3	1.1	1.0–1.2	0.1						
t.l./v.a.l.	11	1.0	0.7–1.2	0.1	7	1.0		3	1.0	1.0	0.0						
C'	11	5.9	5.0–7.5	0.7	1	10.0		3	6.3	5.8–6.7	0.5						
v.a.l./an.b.w.	11	6.1	4.6–7.3	0.7	7	9.7		3	6.6	6.3–6.9	0.3						

*Measurements from Ethiopia, Bulgaria, and Namibia were from Jacobs (1987), Gerlach (1951), and Heyns and Coomans (1989), respectively*.

*A, body length divided by largest body width; a.f.b.l., distance between anterior body end and anterior margin of fovea apertures, measured along the central body axis, an.b.w., anal body width; b, body length divided by pharynx length; c, body length divided by tail length; C', tail length divided by anal body length; c.b.w., body width at pharyngo-cardial junction; f.d., outer fovea aperture diameter measured parallel to body axis; G, distance between ovarium tip and vulva as percentage of body length; h.d., head diameter = body width at the cephalic setae measured perpendicular to central body axis; L, body length measured along central body axis; n, number of specimens measured; n.r., distance between anterior body end and nerve ring as percentage of pharynx length; ph.l., pharynx length; ph.v.l., distance between pharynx and vulva; r.l., rectum length; s, standard deviation; t.l., tail length; v, distance between anterior body end and vulva; V, distance of vulva from anterior body end as percentage of body length; v.a.l., distance between vulva and anus; v.b.w., body width at vulva*.

**Figure 1:**
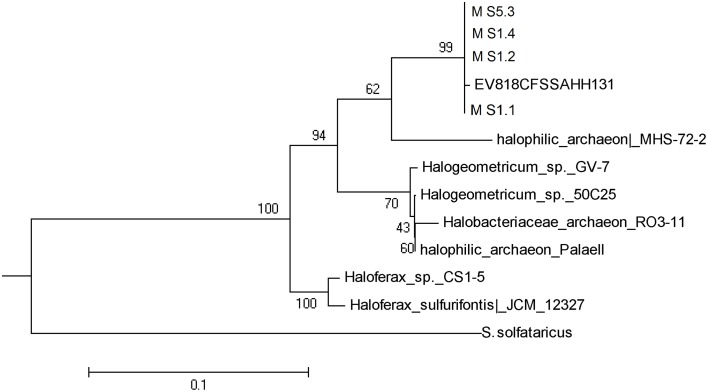
**Phylogenetic tree of the Archaea extracted from the salt stalactites**.

Only the salt stalactite of Moab Khotsong contained 16S rRNA gene sequences belonging to the *Euryarchaeota (Halobacteriales)*, in all 4 dissolved layers (Table 1, Figure 1). NCBI BLAST results of those four sequences showed 99% of identity to one uncultured archaeon clone (EV818CFSSAHH131—*Halobacteriales*) previously detected in a calcitic mineral fracture of a drill core collected from Evander mine level 18 of #8 shaft at a depth of 2 km (Davidson et al., 2011).

**Figure 2:**
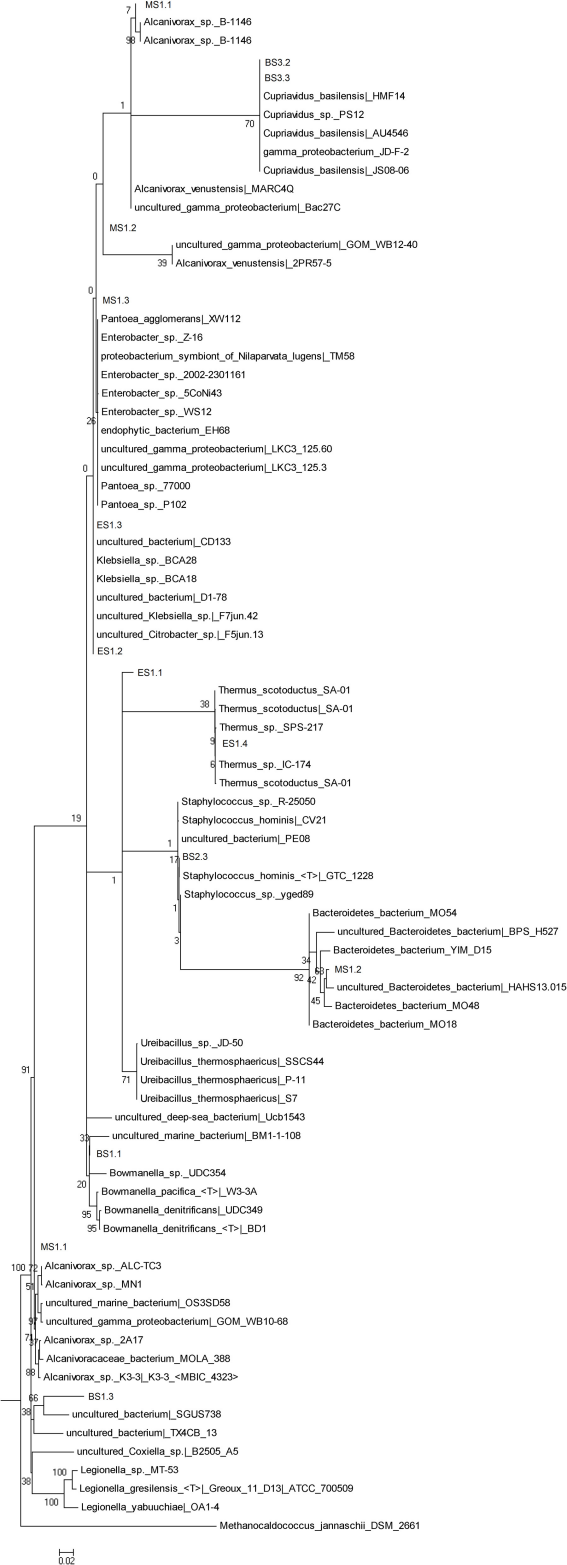
**Phylogenetic tree of the Bacteria extracted from the salt stalactites**.

The bacterial phylogenetic diversity is represented by seven orders (Figure 2). Sphingobacteriales, Thermales, Burkholderiales, Alteromonadales, Enterobacteriales, Oceanospirillales, Bacillales. The largest represented groups were from the Orders Enterobacteriales, Burkholderiales, and Oceanospirillales. Significant was the identification of most likely marine Bacteria. The Order Oceanospirillales included one sequence that shared the closest identity 92% to one uncultured bacterium (SGUS738) identified from coral fragments taken in Panama. Two additional sequences clearly linked to Gamma-proteobacteria and Alcanivorax sp. In the Order Alteromonadales the sequence was closely related to Bowmanella as well as an uncultured marine bacterium. Attempts to culture Archaea and Bacteria from the layered extracts failed on three attempts.

### Culture tests

To support the potential for a marine origin for and to determine the degree of obligatory salt requirement of *M. parvella* we tested the culture in a salt gradient. The salt tolerance test clearly showed a salinity requirement range of 9–14 g/L for *M. parvella* whereas the control species clearly showed very little tolerance for any salt (Table 3). *M. parvella* was difficult to keep in culture since the nematodes were not producing offspring and becoming immobile. In an attempt to halt the decline of the culture an attempt was made to identify the Bacteria associated with the nematodes. One nematode specimen from the culture was sacrificed and DNA sequencing identified the presence of *Alicyclobacillus* sp. (99% identity). This is a spore-forming, aerobic sulfur and ferrous iron oxidizer (*Firmicutes*) (Wisotzkey et al., 1992). Subsequent addition of sulfate (1 mM MgSO_4_ final concentration) to the nematode culture led to a marked rebound of nematode activity however the effect could not be sustained and the nematode culture died out eventually.

**Table 3:** **Salt requirement experiment**.

**Salt g/l**	**0**	**2**	**3**	**5**	**8**	**9**	**10**	**14**	**15**	**16**
*Poikilolaimus oxycercus*	+	+	–	–	–	–	–	–	–	–
*Monhystrella parvella*	–	–	–	–	–	+	+	+	–	–
*Halicephalobus mephisto*	+	+	+	–	–	–	–	–	–	–

*Poiukilolaimus oxycercus is a nematode from the surface, Halicephalobus mephisto a deep subsurface collected species. All are bacteriophagous nematodes*.

### CT scanning

**Figure 3:**
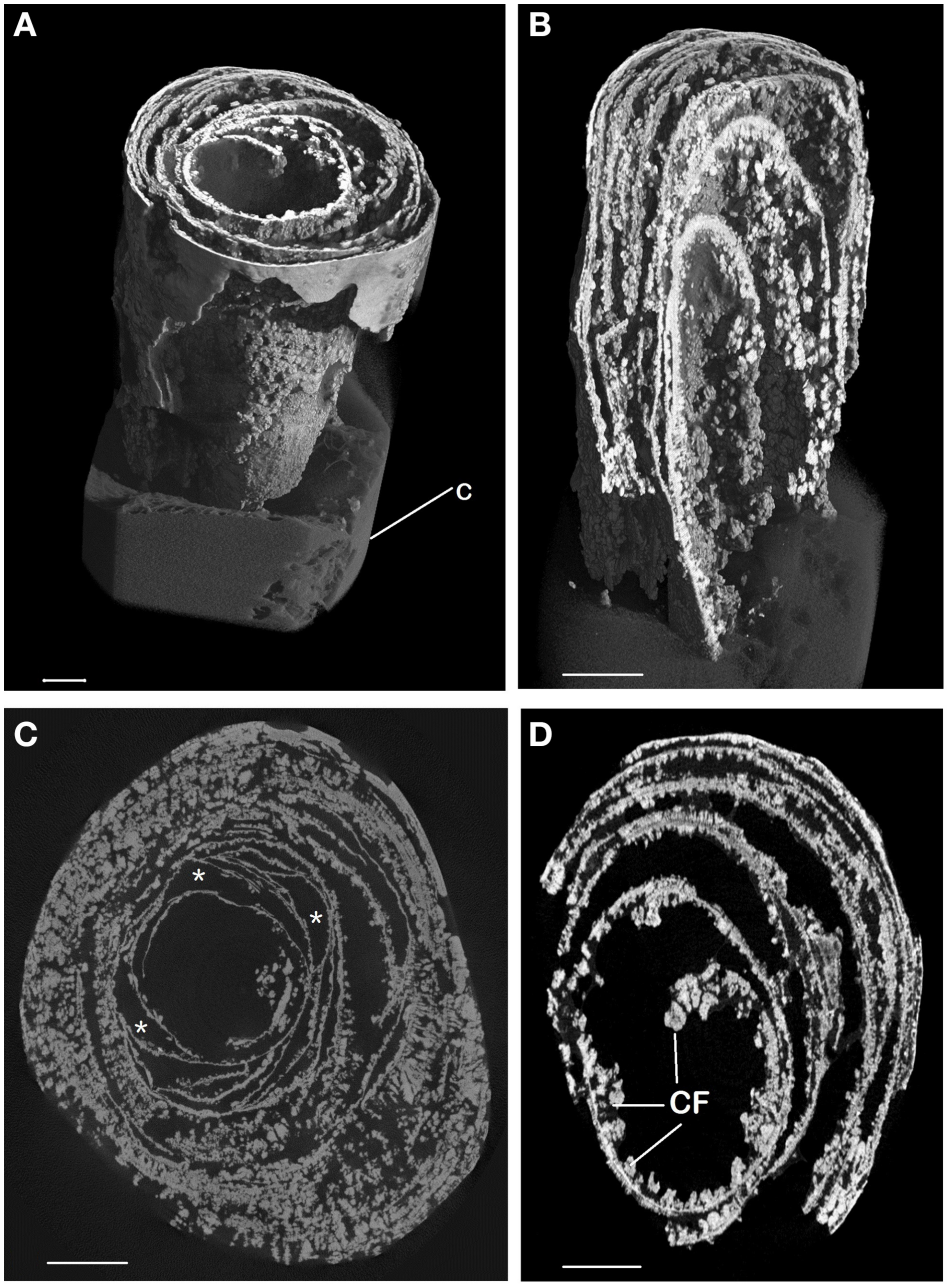
**Micro CT scan of CaCO_3_ stalactite**. **(A)** General habitus of CT scan of CaCO_3_ stalactite that contained nematodes. The area shown is near the tip of the soda straw at the bottom of the stalactite away from the ceiling. The bent laminae are clearly visible as is the knob like outgrowths on them. Cotton (C) was used to stabilize the stalactite for scanning. **(B)** Longitudinal section inside the lower part of image **(A)**. The lamellar built of the stalactite and the outgrowths are plainly visible. **(C,D)** Cross sections of the stalactite in an area close to the ceiling **(C)** and near the end **(D)**. Outgrowths can become very dominant and the laminae can form complete or partially enclosed spaces (^*^) within the inner stalactite. Scale bars: **(A)** 4 cm, **(B)**: 0.1 cm, **(C)**: 7cm, **(D)**: 5 cm.

**Figure 4:**
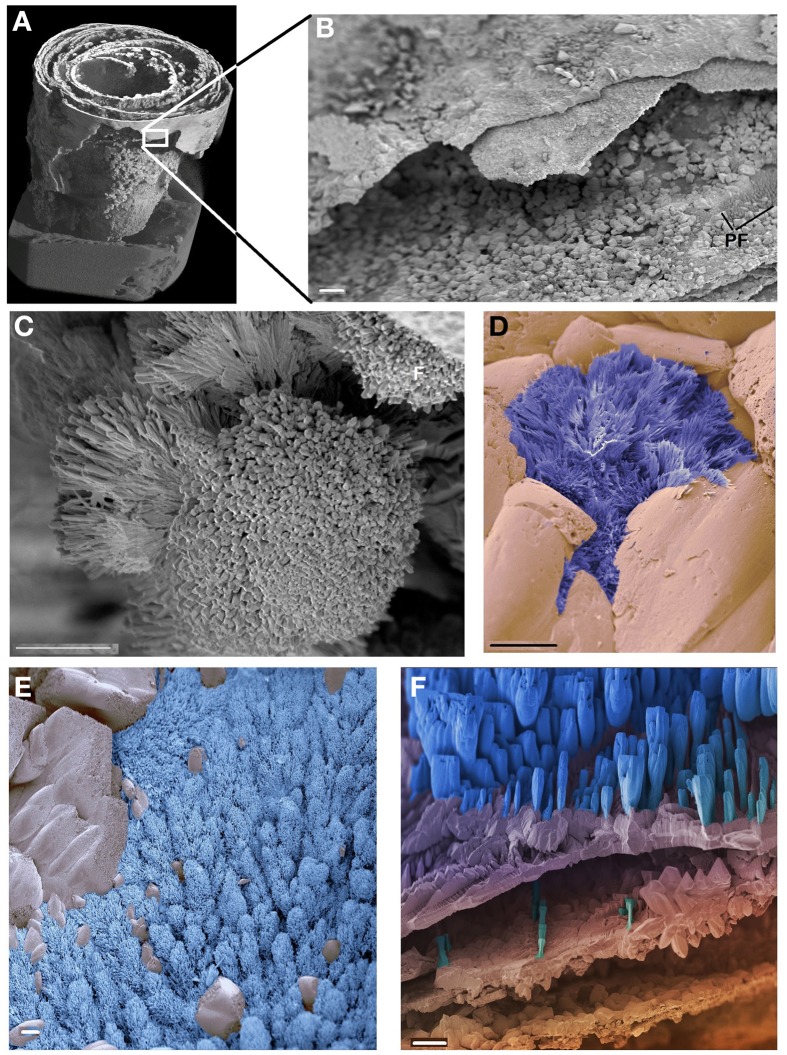
**SEM of the CaCO_3_ stalactite**. **(A)** General built. Image identical to Figure 3A but non-linearly compacted as it only serves to highlight the position of image **(B)**. **(B)** Detail of the outer, smooth surface of the stalactite showing the much coarser built of the more interior layers inside. Low resolution of an area covered in the shrub called “pine forest.” **(C–F)** The different types of shrubs identified in the CaCO_3_ stalactite. **(C)** The “cauliflower” the most dominantly present. **(D)** The “Chinese fan” (dark blue) **(E)** the “pine forest” (light blue). **(F)** The “amphora” (blue). No logical pattern of presence could be deduced for any of these shrubs. Scale bars **(B)** and **(F)**: 100 μm, **(C–E)**: 10 μm.

**Figure 5:**
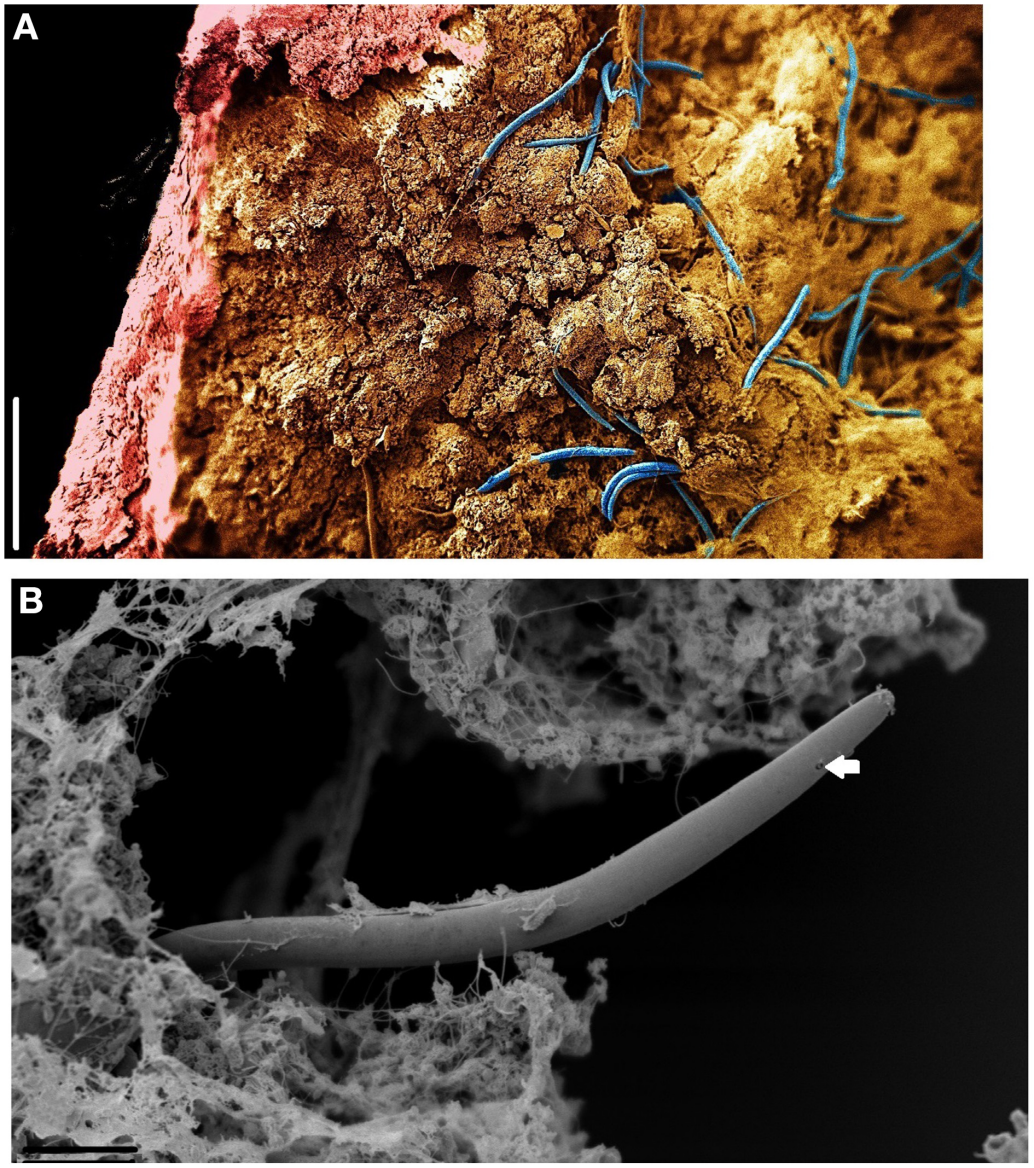
**SEM of ***Monhystrella parvella*** inside a CaCO_3_ stalactite after longitudinal sections with a scalpel**. **(A)** Composite image made of four images taken at different focal points and assembled using Adobe Photoshop. Nematodes (blue) reside in the amorph mass within the confines of the CaCO_3_ outer stalactite wall (pink). **(B)** Detail of *M. parvella*, arrow highlights the fovea. Scale bars: **(A,B)**: 200 μm.

Micro CT scanning and SEM of the CaCO_3_ stalactites that yielded nematodes failed to reveal any apertures in the outermost CaCO_3_ layer. Micro CT scanning revealed a circular layered internal stalactite structure, laminae, with cavities between the layers themselves populated with several type of different shaped outgrowths mainly, although not always, pointed toward the center (Figures 3A–C, Video 1). All CaCO_3_ stalactites scanned showed turning laminae more numerous closer to the ceiling than at the bottom. Using CT scan the highest number of laminae counted was 16 layers of partial or complete laminae in the stalactite part near the ceiling, diminishing to a minimum of 8 near the exit of the straw. Four morphologically different outgrowths were identified; the “coralloid,” the “pine,” the “amphora” and the “Chinese fan” (Figures 3D, 4A–F). Nematodes were identified using SEM between these calcified layers (Figures 5A,B) thereby confirming the nematodes also live inside the stalactite and not only in the central tube of the stalactite.

Macro CT scan of the salt stalactites showed no organized internal structure was evident as in the CaCO_3_ stalactites. To ascertain that this was not due to resolution problems a CT scan of a large salt stalactite from the same sampling site at Moab Khotsong (23 cm length, largest width 8.5 cm weight 2.0 kg) was scanned under a hospital CT-scanner (Videos 2, 3). The scans did not reveal an organized internal structure but a random sponge-like structure punctuated by a considerable number of air/water vacuoles (Videos 2, 3).

## Discussion

Using ethanol-flame sterilized stalactites, extraction of archaeal, bacterial and eukaryotic DNA from salt and CaCO_3_ stalactites is possible. Salt has been shown to be a very effective preservative for DNA (Frantzen et al., 1998) and probably explains the higher diversity yield in salt stalactites. Because the CaCO_3_ stalactites were dry at the moment of sterilization it may explain the less consistent and lower yield which probably originates from organisms in survival stages hence yielding more sequenceable DNA. The drying out would explain the absence of nematode DNA as *M. parvella* does not have a survival stage.

Eumetazoa have been reported inhabiting stalactite like structures (so called biostalactites) (Sanfilippo et al., 2014). But these biostalactites consist of crusts produced by (among others) the Eumetazoa themselves. Generally these are a few centimeters thick and consist of small serpulids and other Eumetazoa. The larger ones often include a nucleus of serpulid tubes. The metazoans include mainly serpuloideans, sponges, bryozoans and foraminifers (Sanfilippo et al., 2014). Nematodes have never been reported living inside stalactites before. Additionally nematodes are not tube building Eumetazoa like e.g., some Annelida. The discovery cannot be compared to the aforementioned biostalactites as it concerns Eumetazoa effectively “trapped” and surviving inside a solid stalactite. The nematodes themselves do not excrete stalactite material or otherwise contribute directly to the stalactite formation.

Puzzling findings were one Archaea and two stalactite Bacteria with a high identity to marine species. MS1.1–1.4 all contained a species with closest identity to *Halobacteriales*. These organisms are the dominant taxa in hypersaline ecosystems, such as salterns, salt and soda lakes and coastal areas, in which NaCl concentrations can reach 150–350 g/L (Andam et al., 2012). However, *Halobacteriales* strains from ecosystems with low salinities (1–3.5% NaCl) have been reported, where they appear to possess exceptional survival capabilities and/or are capable of exploiting niches of relatively higher salinities created due to temporal and spatial variations in geochemical conditions in such ecosystems (Youssef et al., 2012). BS2.1 closest identity to *Bowmanella* sp. is only known from marine habitats. BS2.3 was identified as an uncultured bacterium showing highest similarity again to the marine group *Oceanospirillales*. MS3.1 shared closest identity to *Alcanivorax* sp. also of marine origin (Yakimov et al., 1998). Although the DNA results of the Bacteria are not extensive enough to state as a certainty that these are marine Bacteria, the discovery of *M. parvella* that cannot survive outside a salty environment however does point strongly to a marine fingerprint. Since none of the Bacteria from the stalactites could be cultured the evidence rests on the nematode. Although *M. parvella* resulting from human contamination is difficult to fathom for an organism that requires a salty environment, it is essential to establish that *M. parvella* is indigenous to the stalactite as a result of fissure water. Three major questions are important to determine whether the nematode *M. parvella* recovered is indigenous to the carbonate stalactite: (1) are there apertures in the stalactite that would explain entry in the stalactite by nematodes other than the fissure water?, (2) Is there evidence of bacterial growth serving as a food source for the nematode inside the stalactites?, (3) The high salt tolerance of the nematode species might support indigenous origin, but to what extent is the salt tolerance of the nematode species restrictive?

### Apertures other than the soda straw in the carbonate stalactites

An alternative way of nematode entry would be that a nematode either from the mine tunnel or flowing out of a fissure and into the mine tunnel floor crawls all the way up the wall, on the ceiling where it has to withstand the water flow forming the stalactite and crawl into the stalactite. (Micro)-CT scan and SEM did not reveal any apertures in the carbonate stalactites that could explain an alternative pathway of entry for the nematodes than transport via the fissure water that built the stalactite. Although the absence of apertures does not rule out the gradual “incorporation” of the nematodes as the stalactite grows and nematodes crawl their way up and move against the water drip into the soda straw. Using the Andrassy formula (Andrassy, 1956) and a density of 1.13 g cm^−3^, the estimated dry weight mass of *M. parvella* is 2.03 × 10^−8^ g, which makes it highly unlikely it would be able to move against the water drip on the ceiling. No nematodes were found immediately beneath or in the vicinity of the stalactite drip on the second stalactite collection trip in 2010 or anywhere in the tunnel thus weakening the alternative theory of nematodes crawling up the wall but supporting an indigenous origin of the nematodes.

### Bacteria growth inside carbonate stalactites

If, as we contend, the nematodes become trapped inside the stalactite with the water that forms the stalactite, the nematodes can only survive if they have a ready supply of bacterial food inside the stalactite. Although a number of Bacteria have been identified in the stalactite layers the presence of Bacteria in itself does not constitute evidence that these Bacteria are available as a food source for the nematodes. SEM was used to identify structures that are bacterially induced and support Bacteria were effectively growing inside the CaCO_3_ stalactites. We had therefore, we had to rely on secondary structures indicating the presence of Bacteria the so-called bacterial deposited “shrubs” as described and experimentally grown and proven to be Bacteria induced by Sánchez-Navas et al. (2013). The SEM revealed, based on their morphology, four types of shrubs of which one was very similar to the experimentally induced ones (Figure 14 in Sánchez-Navas et al., 2013). Since we based the types of shrubs on morphology and cannot link a specific type of shrub to a specific Bacteria species. We cannot determine whether the different types of shrubs are just different stages of the same bacterial species or represent different morphologies caused by the growth of different species of Bacteria. Morphologically the large number of “coralloid” shrubs observed, which resemble most closely those experimentally grown and (Sánchez-Navas et al., 2013), indicates a high bacterial growth within the carbonate stalactites and thus food support for nematodes.

### High salt requirement of *M. parvella* supports an indigenous origin

Salt requirement tests showed *M. parvella* could not survive longer than 2 h when salinity in the medium dropped below 9 g/L and did not tolerate salinity higher than 14 g/L. The inability to survive in a salt-less environment in a terrestrial environment is surprising. This test effectively rules out the possibility of *M. parvella* crawling up to the ceiling to the stalactite as none of the water tested in the tunnel has enough salinity to let it survive, besides the observation that it was not found in control samples. The only saline water anywhere near the sampling site where this *M. parvella* could survive is the fissure water that forms the stalactite. These facts strongly support an indigenous origin of the nematode in the stalactite and effectively rule out other possibilities (e.g., contamination, anthropogenic or otherwise). Nematodes have been discovered at 1–3.8 km below the surface in fissure water before (Borgonie et al., 2011), but these were terrestrial/freshwater species. The discovery that brackish water/marine nematode species are surviving in deep underground stalactites in a terrestrial area deep inland is a novelty.

There was initial apprehension that DNA isolated from stalactites in mines would be all “contamination” from mine operations. Specifically an earlier study in a mine environment had indicated a high level of fungal spores in mine air (Pohl et al., 2007). Results of bacterial diversity analysis allow us to broadly distinguish between organisms that can be considered originating from deep mine fissure water and organisms originating from anthropogenic influences. Examples of confirmed deep fissure Archaea/Bacteria are MS3.1 with closest identity to a *Halobacteriales* previously recovered from Evander mine. ES1.4 with closest identity to *Thermus* sp. (Kieft et al., 1999). BS2.1 with closest identity to *Bowmanella* sp. is only known from 40 marine habitats, is Gram-negative, heterotrophic sulfate reducer (Klepac-Ceraj et al., 2004) and very probably also deep fissure water related rather than anthropogenic. Similarly, some Bacteria are clearly not originating from the deep subsurface but are most likely anthropogenic in origin mediated by high moisture, heat and ventilation in the mine tunnels; ES1.2 and BS2.2 showed closest homology to *Klebsiella* sp. and BS2.3 *Staphylococcus* sp. The *Alicyclobacillus* sp. identified with *M. parvella* was not recovered in any of the stalactite layers, which may be due to a nematode linked enrichment phenomenon rather than the absence in the stalactite.

Table 1 lists where the Archaea/Bacteria isolated from the stalactites have been observed in other deep subsurface settings. The bacterial content in the stalactite compared with that of fissure water from Beatrix gold mine could only be done using data from a borehole (Be326) approximately 100 m away from the stalactite collection site. In 2011, a new nematode species, *H. mephisto* that was not marine linked was found in fissure water from this borehole (Borgonie et al., 2011). This species, which was used as a control organism for the halotolerance test in this study (Table 3) could not survive even moderate salt concentrations. Thus, the fissure water at the time was at least chemically different from the fissure water that supported *M. parvella*. The bacterial composition analyzed in 2011 revealed none of the Bacteria found in the stalactite (*Thermus, Bowmanella, Cupriavidus, Oceanospirillales*).

Surprisingly only one fungus (*Candida* sp.) was found and three protozoa (*Acanthamoeba, Colpoda*, and an unknown species). Pohl et al. (2007) had already shown in a previous study the abundance of fungi spores in mine air, which can then easily account for the presence of *Candida* sp. in the stalactites. Similarly for *Acanthamoeba* sp. (Khan, 2009) and *Colpoda* sp. (Finlay et al., 2001), which are both ubiquitously distributed in the environment and are known to develop resistant stages in adverse conditions. If in the future these Eukarya would be confirmed originating from the fissure water it would be the first report of Fungi and Protozoa at this depth in the subsurface of South Africa.

The micro CT scan revealed a highly unusual inner structure of the CaCO_3_ stalactites consisting of numerous laminae with spaces in between and is identical to the structure reported by Martinez-Martinez et al. (2010) for similar stalactites. However, no information as to its genesis was offered nor can we propose one based on our results. SEM analysis (Figure 5) clearly shows that the space between the lamellae is the nematode residing space. This geometry additionally explains the different DNA extracted patterns observed between CaCO_3_ and salt stalactites; salt stalactites form a continuous unit while CaCO_3_ stalactites are divided through laminae.

The current recharge source for Beatrix gold mine is impossible to trace with certainty. Data from the closest borehole (Be326) sampled approximately 100 m away from the stalactites in Beatrix gold mine yields δ^18^O and δ^2^H values (residence time >40–80 kyr) for the fissure water samples that are similar to those previously reported from the Welkom mining region. Although these values are also on the Global Meteoric Water Line (Lau et al., 2014), they are distinct from those lying on the meteoric waterline from the northern and eastern margins of the Witwatersrand Basin (Ward et al., 2004; Onstott et al., 2006). Only two regions exist in South Africa with predicted δ^2^H-values for precipitation that coincide with the very light −40 to −47‰ range observed for the Welkom fracture water samples, the Kalahari Desert and the Lesotho highlands (West et al., 2014). Given that the Welkom mining district lies south of the Vaal River at an elevation of 1370 m, it is more likely that groundwater recharge for this mining district occurs 150 km to the southeast in the mountains of Lesotho (at an elevation of ~2500 m), than from the Kalahari Desert (across the Vaal River to the north and at a lower elevation). The long flow path from the Lesotho highlands could explain the age for the Welkom fracture water (Lau et al., 2014). It is obvious that neither of these freshwater sources can be the source of a salt requiring nematode, Bacteria and/or Archaea species.

### The origin of *M. parvella*

The origin of *M. parvella* is thus enigmatic. The possibility that *M. parvella* was originally a freshwater nematode is unlikely as it would imply that evolution toward obligate brackish water conditions for survival occurred several times independently from each other (Ethiopia (18,g/L), Namibia (salinity not reported, Bulgaria/Black Sea 18 g/L and in the mines (14 g/L). This focuses the origin on marine/brackish water related source on the surface. The geological history of Southern Africa indicates two large windows of opportunity; one 350–250 myr ago during the initial existence stage of the inland Karoo Sea (McCarthy and Rubidge, 2005) and a second geological more recent period linked to the origin of the salt pans in South Africa (Roelofse, 2010). In the event of the Karoo Sea origin, the nematodes could have traveled to the deep subsurface from brackish coastal waters. From the nematode evolutionary timeline there is no conflict; the line that led to the Nematoda split off from the branch to man about 1.1 billion years ago based on histone and cytochrome c sequences (Vanfleteren et al., 1990). The most recent common ancestor to the extant nematode species *Caenorhabditis elegans, Trichostrongylus colubriformis, Nippostrongylus brasiliensis, Ascaris suum*, and *Pseudaterranova decipiens* lived about 550 myr ago based on cytochrome c and globin amino acid sequences (Vanfleteren et al., 1994). Thus, the Precambrium origin of nematodes predates the Karoo Sea by some 200 myr. This, however, does not prove that *M. parvella* as a species already existed at the time of the Karoo Sea and in absence of any decent fossil record, this point remains unsolvable. We were also unable to identify clearly marine sediments (carbonate deposits) overlying the Witwatersrand basin in support of this possibility.

South Africa has a very high number of salt pans per area (Allan et al., 1995). Salt pans are regularly visited by large flocks of birds and this constitutes a way *M. parvella* could have been transported from coastal areas to the inland after the disappearance of the Karoo Sea, as nematode transport by birds has been documented (Frisch et al., 2007). The oldest date for a salt pan known to us in South Africa was cited by Roelofse (2010) as 100 kyr. It is doubtful that this is the upper limit for the presence of salt pans in South Africa but in the absence of more rigid age data on salt pans, this second window of opportunity cannot be more exactly defined other than at least 100 kyr.

Although the possibility of a descent to the deep subsurface by *M. parvella* during the Permian must seem preposterous, there are two arguments in its favor. Firstly, to date and to our knowledge no *M. parvella* has been recovered from the ocean coast but has only been recovered from terrestrial thermal springs (Gerlach, 1951; Jacobs, 1987; Heyns and Coomans, 1989). This argues against the salt pan theory. Second, during the mixed stage culture attempt of *M. parvella*, it became obvious that no survival stage (dauer) was formed. This indicates this species does not have a way to survive unfavorable conditions. Furthermore, the culture of *M. parvella* showed an absolute requirement of salinity for its survival. This means that *M. parvella* must have been in continuous salty water during its entire descent to the deep subsurface. Any mixing with fresh water that diluted the salt too much would have been deadly. Therefore, if the area of descent is covered by a sea, as it was during the Permian/Triassic, groundwater would have been salty and offers a higher probability for *M. parvella* to remain in suitable brackish water. If the area of descent is pockmarked by salt pans as a source of salty groundwater but the majority of the area yields fresh groundwater the chances for continuous suitable salty conditions are less.

Unfortunately we only have DNA sequences of the subsurface populations of *M. parvella*, none from the known surface populations. Therefore, we cannot use DNA, for now, as a marker to determine to what extent both populations have diverged and hence support either window of opportunity.

## Conclusion

This study has demonstrated that stalactites can be used to extract valuable information on Archaea, Bacteria, and Eukarya even after the water flow that formed the stalactites has ceased. This trapping/concentrating function is all the more evident if one compares the DNA yield/number of individual species extracted from the small pieces of stalactite vs. the huge amounts of fissure water that is normally filtered to obtain bio signatures (e.g., Lau et al., 2014). It is unclear how long the environment inside the stalactite remains viable for eumetazoa once the fissure flow stops. If the species have a survival stage in their life cycle then renewed flow at a later date could reactivate them. The stalactites used in this study were small and were uniform in morphology. Future research may determine whether it would be possible to distinguish if the characteristics of different water flows over time can be determined from individual stalactite layers. Contamination due to anthropogenic activity is present but offers in itself new possibilities for archeologists as an additional tool to study human inhabited caves.

### Conflict of interest statement

The authors declare that the research was conducted in the absence of any commercial or financial relationships that could be construed as a potential conflict of interest.

## Supplementary material

Video 1 **Micro CT scan of CaCO_3_ stalactite**.

Video 2 **Macro CT scan of large salt stalactite from Moab Khotsong showing the many randomly dispersed vacuoles (black) inside**.

Video 3 **CT scan cross sections of the salt stalactite from Moab Khotsong**. Several vacuoles can be followed along the reconstruction, clearly showing the enclosed nature of several of these.
